# Transcriptomic Profiling Reveals 17β‐Estradiol Treatment Represses Ubiquitin‐Proteasomal Mediators in Skeletal Muscle of Ovariectomized Mice

**DOI:** 10.1002/jcsm.13698

**Published:** 2025-01-25

**Authors:** Georgios Kararigas, Mara C. Ebeling, Gengyun Le, Shaojuan Lai, Chunmei Cui, Qinghua Cui, Dawn A. Lowe

**Affiliations:** ^1^ Department of Physiology, Faculty of Medicine University of Iceland Reykjavik Iceland; ^2^ Division of Physical Therapy and Rehabilitation Science, Department of Family Medicine and Community Health University of Minnesota Minneapolis Minnesota USA; ^3^ College of Basic Medicine Guizhou University Guiyang Guizhou China; ^4^ Department of Biomedical Informatics, State Key Laboratory of Vascular Homeostasis and Remodeling, School of Basic Medical Sciences Peking University Beijing China; ^5^ School of Sports Medicine Wuhan Institute of Physical Education Wuhan China

**Keywords:** aging, estrogen, estrogen receptor α, estrogen response element

## Abstract

**Background:**

With a decline of 17β‐estradiol (E2) at menopause, E2 has been implicated in the accompanied loss of skeletal muscle mass and strength. We aimed at characterizing transcriptomic responses of skeletal muscle to E2 in female mice, testing the hypothesis that genes and pathways related to contraction and maintenance of mass are differentially expressed in ovariectomized mice with and without E2 treatment.

**Methods:**

Soleus and tibialis anterior (TA) muscles from C57BL/6 ovariectomized mice treated with placebo (OVX) or E2 (OVX + E2) for 60 days, or from skeletal muscle‐specific ERα knockout (skmERαKO) mice and wild‐type littermates (skmERαWT), were used for genome‐wide expression profiling, quantitative real‐time PCR and immunoblotting. Computational detection of estrogen response elements (EREs) was performed with EREFINDER.

**Results:**

We found 155 significantly regulated probe sets in response to E2 (*p* ≤ 0.001). Pathway analyses identified proteasome and ubiquitin‐mediated proteolysis as two downregulated pathways in the E2 group. We confirmed downregulation (*p* ≤ 0.05) in levels of *Fbxw7*, *Psmb6*, *Ube2h* and *Ubxn1*, as well as pro‐apoptotic *Bnip3* and inflammatory factor *Nfkbia*. Computational analysis identified ERE in the promoter regions of *Psmb6*, *Ube2h*, *Bnip3* and *Nfkbia*. The overall content of ubiquitinated proteins was modestly but significantly lower in TA muscles from OVX + E2 vs. OVX mice (*p* = 0.039). There were no differences between skmERαKO and skmERαWT mice or between skmERαKO/OVX and skmERαKO/OVX + E2 mice for any genes assessed, indicating that ERα is required for E2 regulation of those genes.

**Conclusions:**

These results suggest that a mechanism whereby E2 protects against losses of skeletal muscle mass and strength is regulation of ubiquitin‐proteasomal mediators.

## 1 Introduction

Menopause is associated with frailty [1] and losses of skeletal muscle mass and strength, with the earliest decline occurring during peri‐menopause [2]. Due to the substantial decrease in the serum level of the steroid hormone 17β‐estradiol (E2) at menopause, E2 has been implicated in the regulation of muscle function. Supporting this, skeletal muscles of post‐menopausal women on estrogen‐based hormone therapy are stronger than those of women not on hormone therapy [3, 4, 5], and force generation was greater in single fibres from biopsies of post‐menopausal twin sisters on hormone therapy compared with their twins not on hormone therapy [6]. Similarly, in rodents, the loss of ovarian hormones has detrimental effects on skeletal muscle force‐generating capacities [7, 8] and improved recovery of strength following injury [9]. Further, E2 appears to modulate the phospho‐landscape of skeletal muscle proteins [10], maintain muscle contractility through phosphorylation of myosin regulatory light chain [11] or maintain calcium homeostasis in muscle cells through phosphorylation of the ryanodine receptor [12].

E2 plays a key role in several (patho)physiological processes of a wide range of tissues, regulating the activity of many target genes and signalling cascades through genomic or non‐genomic actions. These E2 actions are primarily mediated by estrogen receptor (ER) α and β, which belong to the steroid hormone receptor superfamily of transcription factors and are expressed in skeletal muscle [13], or by the G‐protein‐coupled receptor (GPER), which has also been identified in skeletal muscle [14].

Although many effects of E2 on skeletal muscle function are recognized (e.g., recently reviewed in [15, 16]), the complex underlying mechanisms are incompletely understood. In particular, the potential E2‐dependent regulation of muscle gene expression is incompletely explored, and additional studies are required to better understand the transcriptomic mechanisms of the E2/ER axis in skeletal muscle. In this process, even though ERα is the predominant ER in skeletal muscle and E2 signals through ERα to affect muscle contractility [17], it is important to confirm the role of ERα in mediating such transcriptomic effects of E2 in skeletal muscle. In the present study, we aimed at characterizing the transcriptomic response of skeletal muscle to E2 treatment in ovariectomized mice, testing the hypothesis that genes and pathways related to contraction and maintenance of mass are regulated by E2.

## 2 Methods

### 2.1 Experimental Animals

Female C57BL/6 mice were purchased from the National Institute on Aging colony at 3 months of age and were ovariectomized at 4 months of age. At the time of ovariectomy, mice were randomly selected to be implanted with placebo pellets (OVX) or pellets designed to release 0.18 mg of E2 over 60 days (OVX + E2). The period of 60 days was selected to represent a chronic condition and thus remodelling of the muscle in response to loss of E2 and specific treatment with E2. The E2 dose was previously shown to lead to physiological serum E2 levels [18]. All mice were kept on a 12–12‐h light/dark cycle in temperature‐controlled rooms with water and food ad libitum. At the end of the 60‐day treatment, soleus and tibialis anterior (TA) muscles were dissected from pentobarbital‐anaesthetized mice (100 mg/kg i.p.), snap‐frozen in liquid nitrogen and stored at −80°C. Uteri were also dissected and weighed. Serum was collected during exsanguination and assayed for E2 by ELISA (RE50241, Immuno Biological Laboratories). To determine the extent that effects of E2 on selected genes were through ER α (ERα), skeletal muscle‐specific ERα knockout (skmERαKO) mice and their wild‐type littermates (skmERαWT) were also employed. Generation and characterization of these mice have been previously described [17]. All animal procedures were approved by the institutional animal care and use committee and complied with international guidelines for animal experimentation.

### 2.2 Hybridization and Microarray Profiling

Total RNA was isolated from soleus and TA muscles using TRI Reagent (Sigma) following the manufacturer's protocol. The RNA quality and quantity were established using a 2100 Bioanalyzer (Agilent Technologies). Biotinylated complementary RNA (cRNA) from soleus muscles was prepared and hybridized to the Mouse Genome 430 2.0 array (Affymetrix) according to the standard Affymetrix processing protocol. The array was scanned in a GeneChip Scanner 3000. The quality of hybridization was assessed in all samples following the manufacturer's recommendations. Microarray data are deposited in the Gene Expression Omnibus database under Accession No. GSE134409.

### 2.3 Microarray Data Analysis

The computational and statistical analysis of the microarray data was carried out using the R Version 2.14.2 software [19] and the Bioconductor packages [20] as described previously [21, 22, 23]. Following background correction, expression data were normalized with the variance stabilization and normalization algorithm [24] and log_2_ transformed using the median polish algorithm of robust multi‐array average [25]. The quality of the data was assessed with the *affy* and the *arrayQualityMetrics* packages. To detect differences in probe set expression between different conditions, a moderated linear model was applied using the *limma* package. Pathway analysis was performed by means of gene set enrichment analysis [26, 27] querying the Kyoto Encyclopedia of Genes and Genomes (KEGG) database [28] and by using the *Category* and *GSEABase* packages. At this step, the complete dataset was subjected to non‐specific filtering with the only criterion that each feature should have an Entrez Gene ID annotation. Furthermore, control probes were removed, and genes represented by more than one probe set were collapsed to the probe set with the largest variability. Following the compilation of gene sets, those with less than 10 annotated genes were excluded from downstream analysis. The distribution was computed based on 1000 permutations, and a threshold of 10% was applied.

### 2.4 Quantitative Real‐Time PCR

Total RNA from TA muscles was reverse transcribed with the VILO cDNA Synthesis Kit (Thermo Fisher Scientific Inc) or iScript cDNA synthesis kit (BioRad), and real‐time PCR was performed using TaqMan Fast Advanced Mastermix (Applied Biosystems) and TaqMan primer probes (Thermo Fisher Scientific) and run on a BioRad C1000 Touch PCR Thermal Cycler with CFX96 Real Time System. Reactions where RNA or reverse transcriptase had been omitted were used as negative controls. The amount of each gene of interest (*Ube2h, Ubxn1, Fbxw7, Psmb6, Bnip3, Nfkbia*) was normalized using the average of the expression of *Gapdh* and *Rna18s1*.

### 2.5 Western Blot

Protein homogenates from soleus and TA muscles were prepared in Kei's buffer (20 mM Tris–HCl, pH 7.4, 5 mM EDTA, 1% IGEPAL) with a Bullet Blender. Protein concentrations were determined with the Bradford Assay using albumin as the standard. Proteins were resolved on SDS‐PAGE gels, transferred to polyvinylidene difluoride (PVDF) membranes and blots were blocked with 5% bovine serum albumin. Membranes were incubated overnight with primary antibodies, ubiquitin (Abcam), proteasome 20S subunit β5 (Psmb5; Thermo Scientific), proteasome 20S subunit alpha7 (Psma7; Enzo Life Sciences) or proteasome 26S subunit, non‐ATPase 11 (Psmd11; Cell Signaling Tech). Dylight‐conjugated secondary antibodies (Cell Signaling Tech) were used to detect immune reactions. Images were taken on a Li‐Cor Odyssey CLx. Densitometry was performed using ImageJ software version 1.54e.

### 2.6 Computational Detection of Estrogen Response Elements (EREs)

EREFINDER [29] was used to identify EREs with default parameters, evaluating binding affinities of ERα in the genome of *Mus musculus* GRCm38. Regions of 5 kb before the transcription start site were employed. Briefly, FASTA sequences were used as input for EREFINDER, which scans ERE motifs. EREFINDER was used with a 15 bp‐wide sliding window, sliding one base at a time to calculate a dissociation constant (Kd). EREs were defined as identified ERE motifs with 1/Kd > 0.09.

### 2.7 Statistical Analysis

Grubb's tests were used to identify single outliers; the only data sets in which a single outlier was removed included Ube2h gene expression, and Psmd11 and Psmb5 protein contents. Comparisons between two groups were made with unpaired *t*‐tests. Data are presented as mean ± SD. Statistical significance was assessed using GraphPad Prism 7 (GraphPad software). *p* ≤ 0.05 was considered significant.

## 3 Results

### 3.1 Serum E2 Levels and Body Mass

**FIGURE 1:**
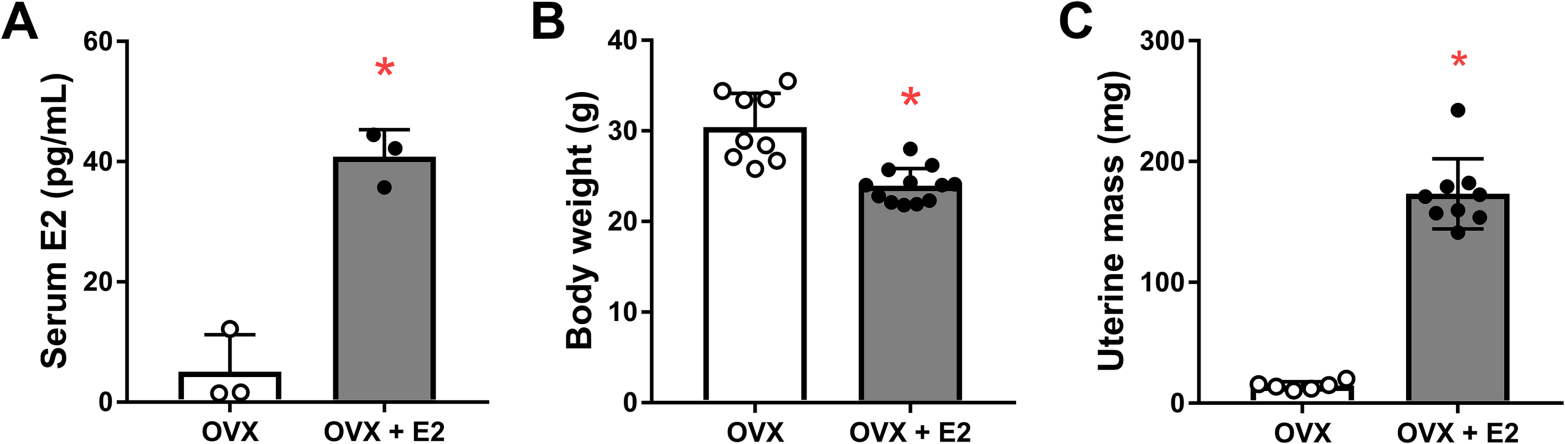
Confirmation of successful hormone treatment. (A) Serum 17β‐estradiol (E2) levels. Body weight (B) and uterine mass (C) in ovariectomized mice (OVX) and ovariectomized mice treated with E2 (OVX + E2) for 60 days. Data are mean ± SD; *p ≤ 0.001.

To verify that the hormone treatment protocol was successful following ovariectomy, circulating E2 levels were measured in a subset of mice. Serum E2 was significantly higher in OVX + E2 mice compared with OVX mice and importantly was measured to be within the physiological range for female mice (*p* = 0.001; Figure 1A). As expected, OVX + E2 mice weighed significantly less (*p* < 0.001; Figure 1B) and had ~12‐fold greater uterine mass (*p* < 0.001; Figure 1C) than OVX mice (treated with placebo) at the end point of the study.

### 3.2 Effects of E2 on Soleus Muscle Transcriptome of Ovariectomized Mice

**FIGURE 2:**
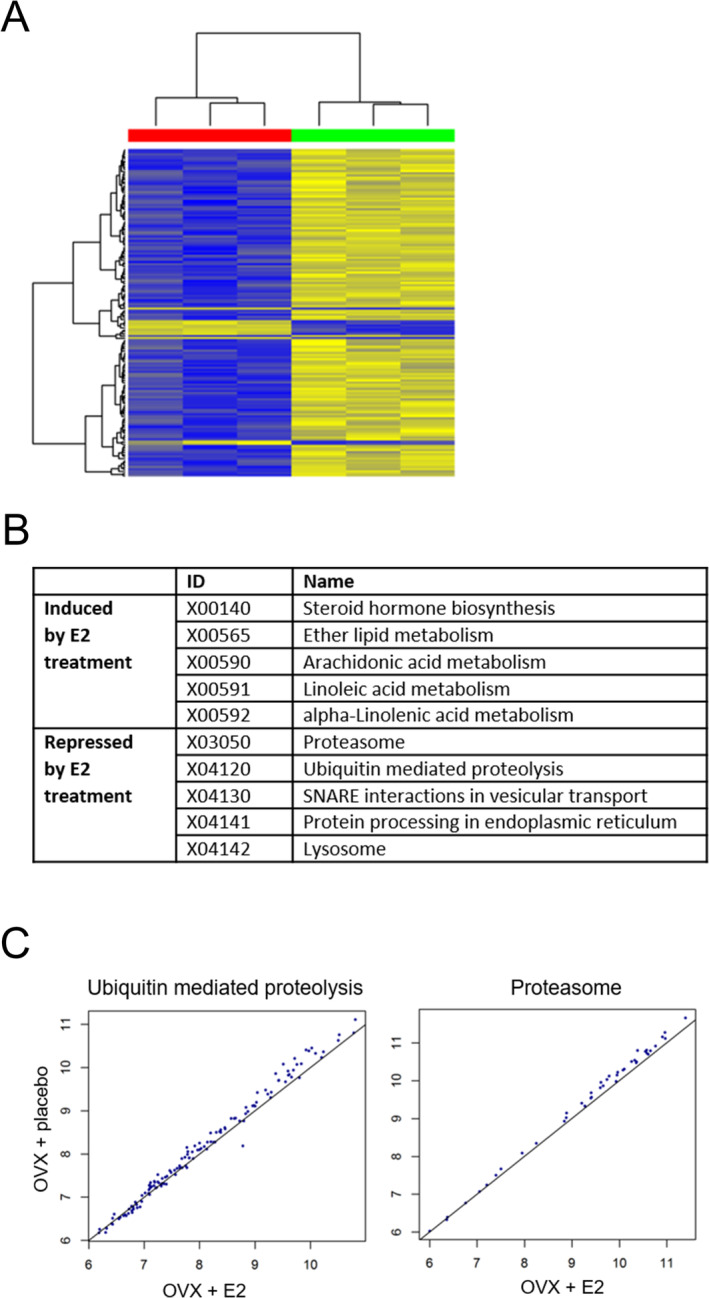
E2‐dependent gene regulation. A. Unsupervised hierarchical clustering of the E2‐dependent differentially expressed probe set expression and the treatment factor of the RNA source. Blue indicates low expression and yellow indicates high expression. In the coloured horizontal side bar, green indicates OVX mice and red indicates OVX mice treated with E2. B. Top 5 altered (induced or repressed) pathways with E2 treatment*.* C. Scatter plots of within‐group means for genes annotated at the ubiquitin‐mediated proteolysis and proteasome pathways. Each point represents an annotated gene. The value on the *x*‐axis is the log2 mean (arbitrary unit) in the E2‐treated OVX mice, whereas the value on the *y*‐axis is the log2 mean (arbitrary unit) of the untreated OVX mice. The diagonal line serves as the divider between groups.

First, to ensure adequate quality of the transcriptomic data, we verified that there were no outliers due to biological or technical reasons. Next, the data for differences in gene expression of soleus muscle between OVX and OVX + E2 mice were analysed by fitting a linear model with an empirical Bayesian method [30]. To extract biologically useful information, those probe sets were examined with an unadjusted *p* value of up to 0.001. This resulted in 155 significantly regulated probe sets (Table S1). Of these, the expression of just 11 probe sets was upregulated, while the remaining 144 were downregulated in the OVX + E2 group compared with the OVX group. Unsupervised hierarchical clustering of the expression profiles for the identified probe sets revealed a distinct E2‐biased gene expression profile, as verified by the separate clustering of the RNA sources based on the treatment annotation and visualized easily by the coloured horizontal side bar (Figure 2A).

### 3.3 Pathway Analysis of the Effects of E2 on Gene Expression in Soleus Muscle of Ovariectomized Mice

Following analysis of single‐gene differential expression, we explored the data further with the gene set enrichment analysis approach [26, 27] using the KEGG [28], aiming at the identification of entire pathways modulated in soleus muscle due to E2. This approach ensured a more comprehensive and biologically integrative analysis of the data. Similar to the analysis at the single‐gene level, the majority of the regulated pathways was downregulated in muscle from OVX + E2 mice compared with those from OVX mice. Specifically, 70 pathways were downregulated in the soleus muscle of E2‐treated mice, while 21 pathways were upregulated (Figure 2B and Table S2). Proteasome and ubiquitin‐mediated proteolysis were two of the downregulated pathways in the E2 group (Figure 2C).

### 3.4 Assessment of Selected Candidates in TA Muscle of Ovariectomized Mice

**FIGURE 3:**
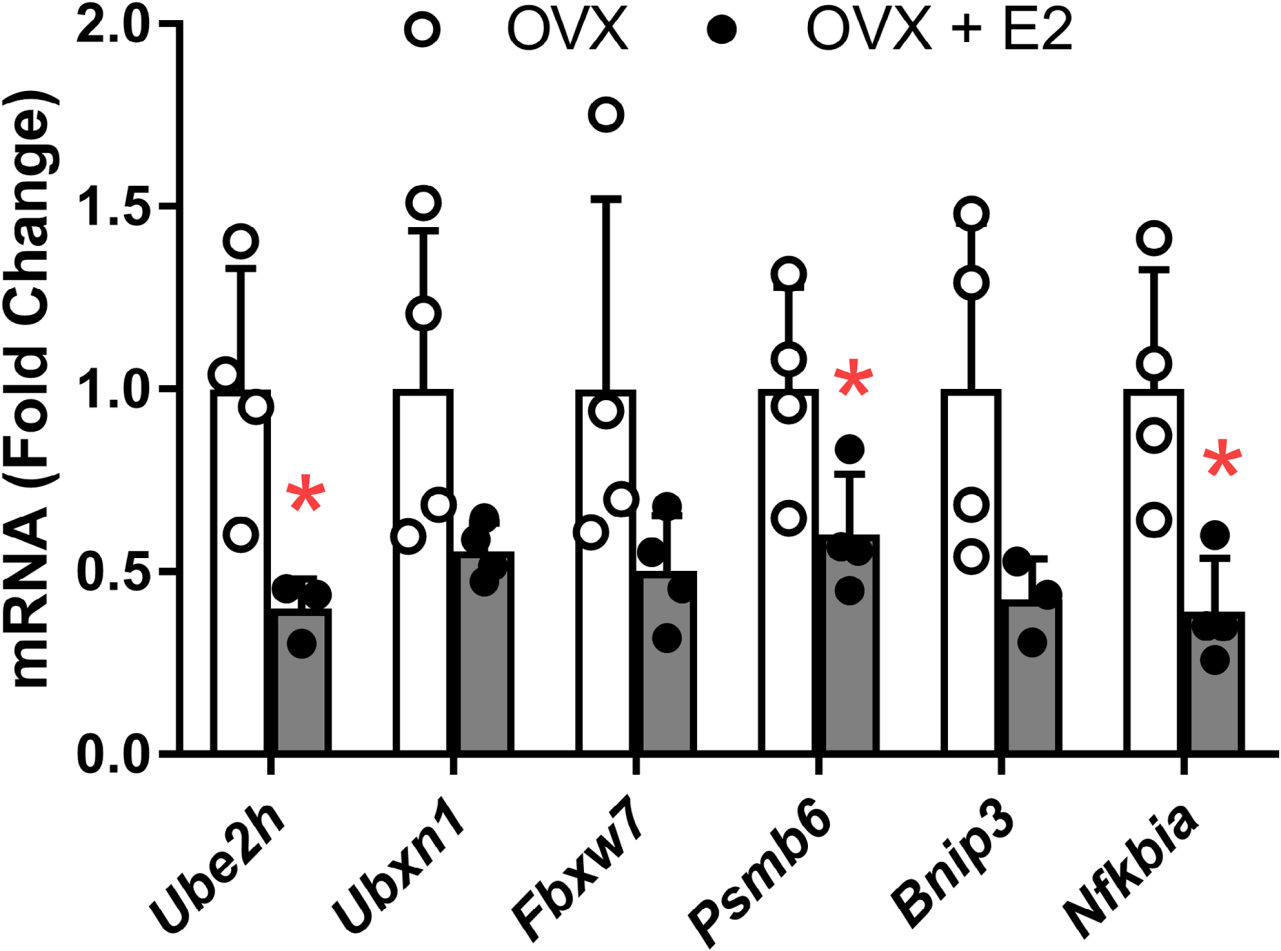
Expression of selected genes by quantitative real‐time RT‐PCR in tibialis anterior muscle of ovariectomized mice (OVX) and ovariectomized mice treated with E2 (OVX + E2). Each data point is a sample from an individual mouse. Data are mean ± SD; *p ≤ 0.05.

To validate the robustness and confirm the biological relevance of our findings, we aimed at assessing whether the E2‐dependent gene regulation in soleus muscle occurs also in TA muscle. Due to the regulation of proteasome and ubiquitin‐mediated proteolysis genes and pathways identified by our whole transcriptome analysis and based on our initial hypothesis, we primarily selected candidates within these biological processes (i.e., *Fbxw7*, *Psmb6*, *Ube2h* and *Ubxn1*), as well as a pro‐apoptotic (*Bnip3*) and an inflammatory factor (*Nfkbia*), for validation of the high‐throughput data by measuring their levels with quantitative real‐time RT‐PCR. In line with the transcriptomic data on soleus muscle, the expression of all selected genes was reduced in the TA muscle of OVX + E2 compared with OVX mice, whereby statistical significance was reached for three of the six genes, that is, *Ube2h*, *Psmb6* and *Nfkbia* (Figure 3).

**FIGURE 4:**
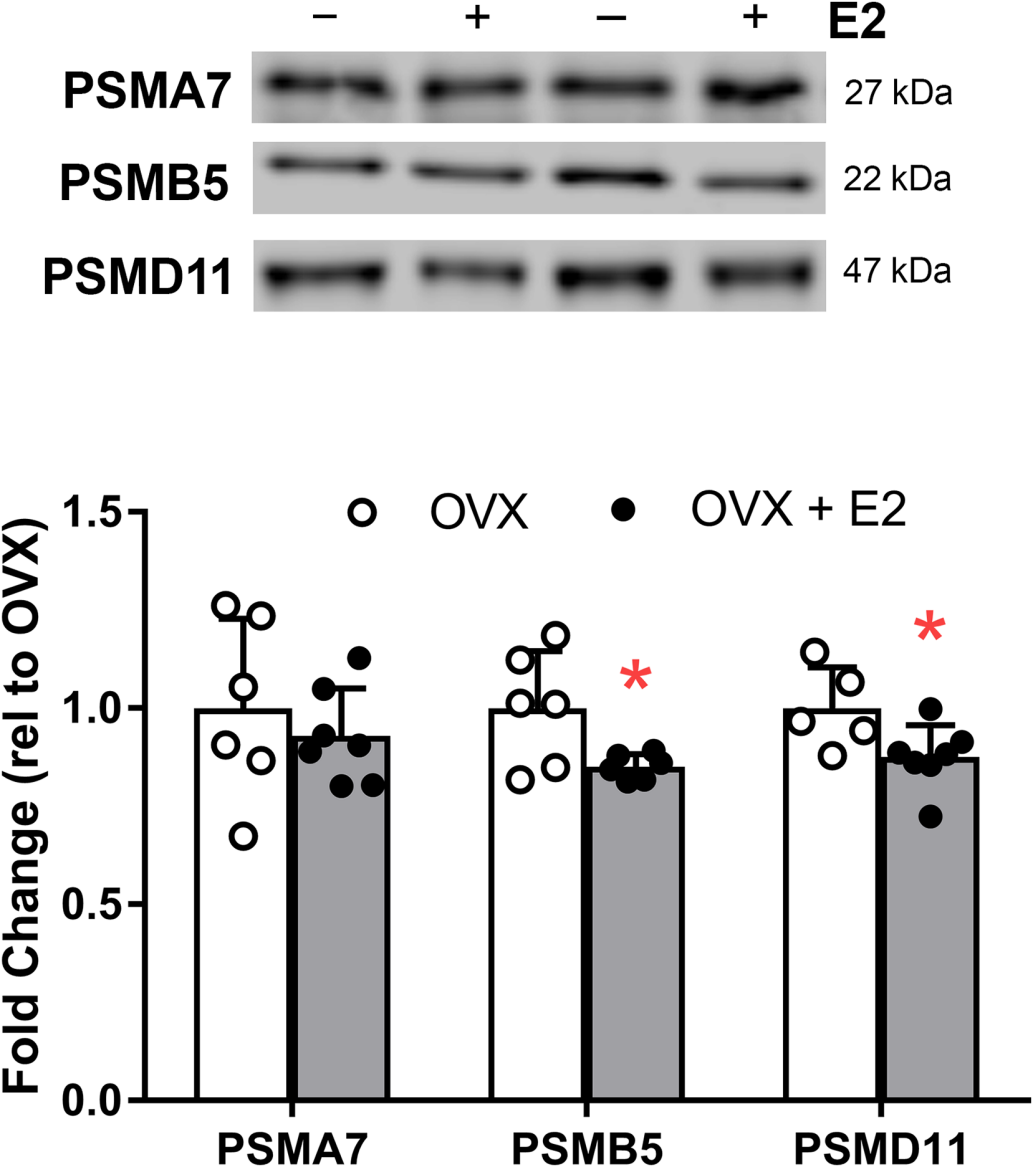
Representative Western blot images and quantification of proteasome‐related proteins in tibialis anterior muscle of ovariectomized mice (OVX) and ovariectomized mice treated with E2 (OVX + E2). Each data point is a sample from an individual mouse. Data are mean ± SD; *p ≤ 0.05.

To determine if the changes detected in mRNA corresponded to changes in protein, the content of three proteasomal proteins was measured by immunoblot. While the content of PSMA7 was not affected by E2 treatment (*p* = 0.593), PSMB5 and PSMD11 were 15% lower in TA muscle from OVX + E2 than OVX mice (*p* ≤ 0.045; Figure 4), supporting the transcriptomic results.

### 3.5 Effects of E2 on Ubiquitination in TA Muscle of Ovariectomized Mice

**FIGURE 5:**
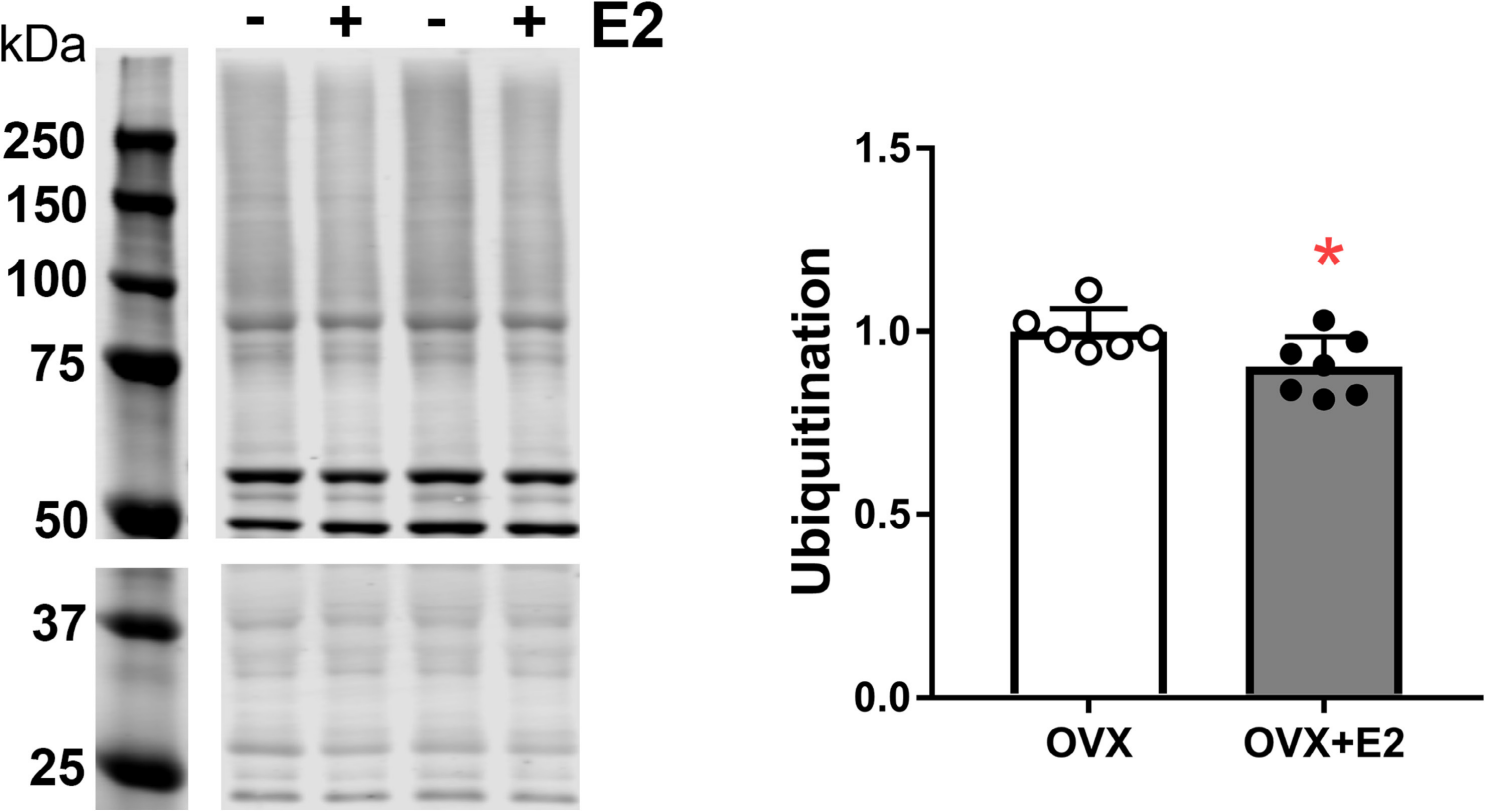
Representative Western blot images and quantification of ubiquitinated proteins in tibialis anterior muscle of ovariectomized mice (OVX) and ovariectomized mice treated with E2 (OVX + E2). Protein bands from 50 to 250 kDa were quantified on 8% gels (top) and bands from 20 to 50 kDa were quantified on 12% gels (bottom). Each data point is a sample from an individual mouse and represents the sum of band densities from 20 to 250 kDa. Data are mean ± SD; *p ≤ 0.05.

Based on the overall repression of ubiquitin‐proteasomal mediators by E2, we hypothesized that total ubiquitination of muscle proteins in OVX + E2 mice would be decreased. Accordingly, the overall content of ubiquitinated proteins was significantly lower in TA muscles from OVX + E2 compared with OVX mice (*p* = 0.039; Figure 5).

### 3.6 Involvement of ERα in the Regulation of Selected Genes

**FIGURE 6:**
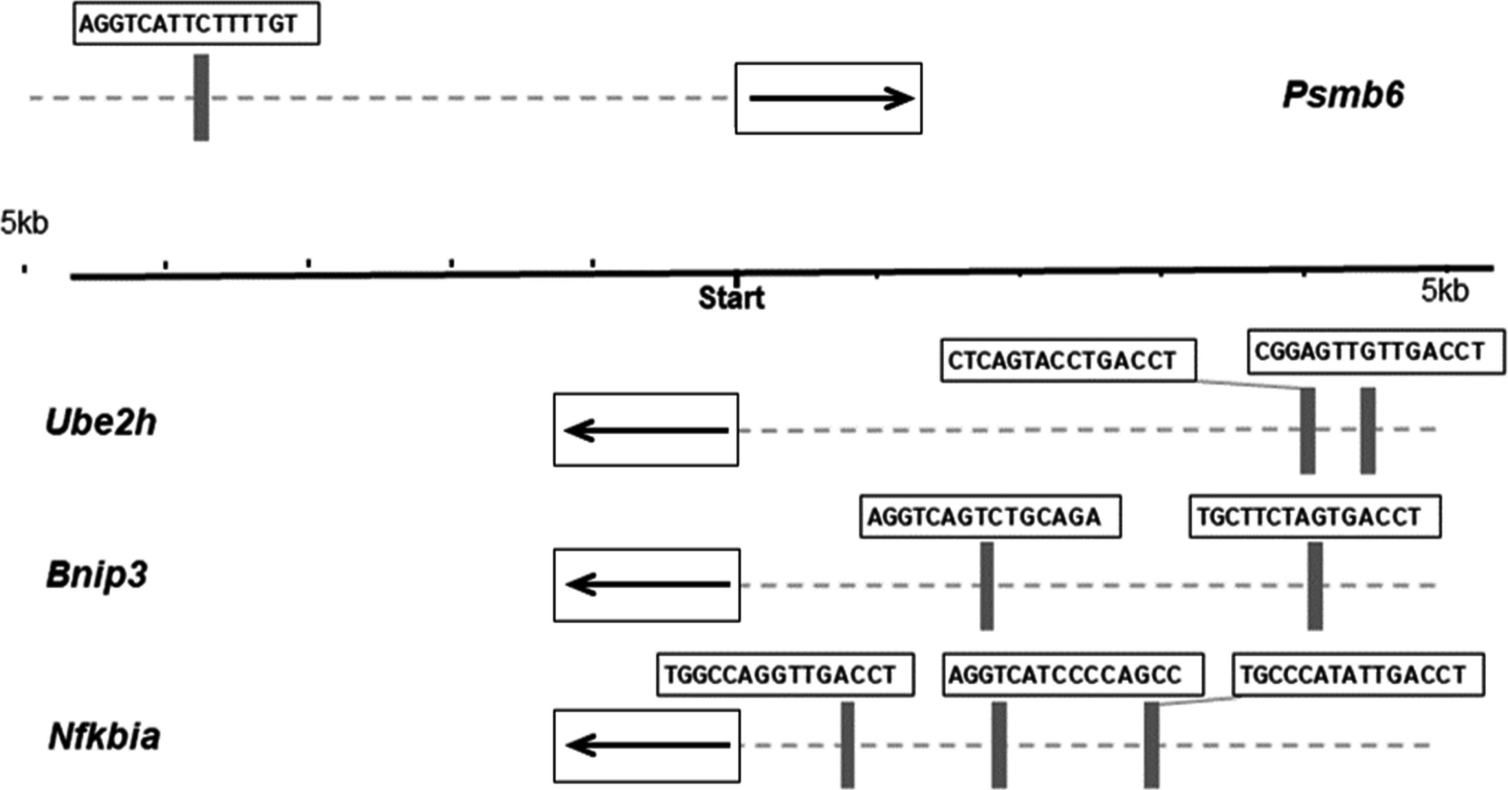
Computational detection of estrogen response elements (EREs). ERE detected in four of six target genes. ER‐binding regions are indicated by the vertical grey rectangles, with the corresponding predicted ER‐binding sequences directly above in white boxes.

On the basis that ERα is the predominant ER in skeletal muscle and that E2 signals through ERα to affect muscle contractility [17], we conducted three experiments to determine the mechanistic extent that the E2/ERα axis is necessary for the regulation of ubiquitin‐ and proteasome‐related mediators. First, we performed a computational analysis for EREs in the promoter region of the selected genes, *Fbxw7*, *Psmb6*, *Ube2h*, *Ubxn1*, *Bnip3* and *Nfkbia*. Our analysis revealed that four of these genes have at least one ERE, that is, *Psmb6*, *Ube2h*, *Bnip3* and *Nfkbia* (Figure 6). In particular, three EREs with high binding affinity were found in the promoter region of *Nfkbia*, two in *Ube2h* and *Bnip3*, and one ERE was found in the promoter region of *Psmb6*. These data indicate that the putative ERE might play an important role in the repression of those genes by E2.

**FIGURE 7:**
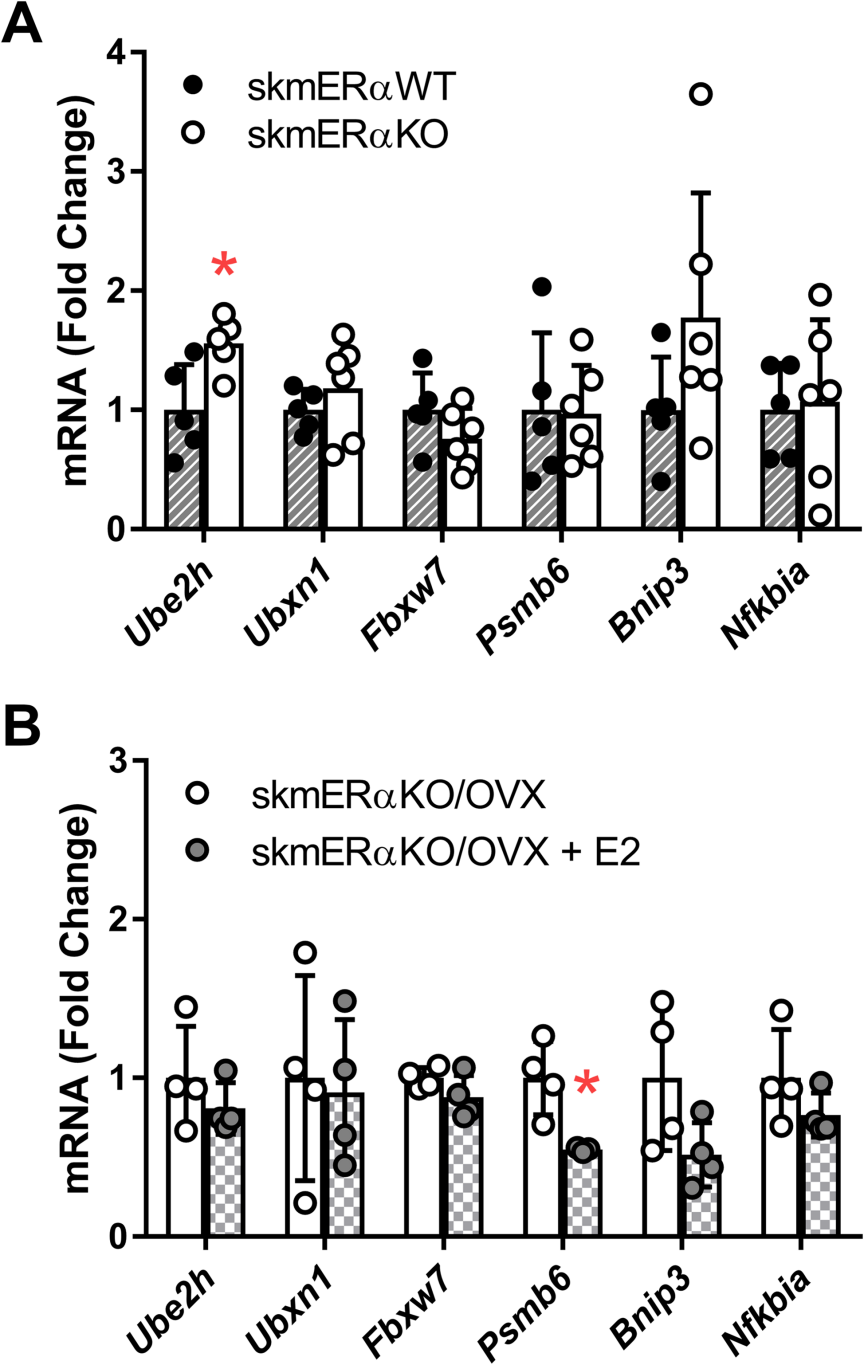
Expression of selected genes by quantitative real‐time RT‐PCR of tibialis anterior muscle from (A) ERα skeletal muscle knockout (skmERαKO) compared with wild‐type (skmERαWT) mice and from (B) skmERαKO mice, ovariectomized and treated with placebo or E2. Each data point is a sample from an individual mouse. Data are mean ± SD; *p ≤ 0.05.

Next, we compared the expression of these six genes in TA muscles of wild‐type (skmERαWT) mice and mice with skeletal muscle‐specific deletion of ERα (skmERαKO), with both groups having their ovaries intact and thus endogenous E2 in circulation. There was no significant difference in the expression of five selected genes between muscle from skmERαWT and skmERαKO mice (Figure 7A). The expression of *Ube2h* was significantly higher in skmERαKO compared with skmERαWT mice (*p* = 0.023). These data provide some additional evidence that the effects of E2 on these genes in skeletal muscle are mediated through ERα.

In our third line of experimentation, skmERαKO mice were ovariectomized and treated with placebo or E2. There was no significant difference between skmERαKO/OVX and skmERαKO/OVX + E2 mice for five of the six genes assessed (Figure 7B). These results provide further evidence that E2 requires ERα to repress the expression of the selected ubiquitin‐proteasome genes in the skeletal muscle of female mice.

## 4 Discussion

In the present study, we investigated the effects of E2 on global gene expression in the skeletal muscle of ovariectomized mice. To our knowledge, this is the first study reporting genome‐wide effects of E2 in the skeletal muscle of female mice, identifying previously unrecognized targets of E2 in this tissue. Importantly, we found that the regulation of selected candidates by E2 occurs in both soleus and TA muscles (Figures 2 and 3). Of note, the expression of the vast majority of the genes and pathways was downregulated in response to the E2 treatment, some of which included the proteasome and ubiquitin‐mediated proteolysis pathways. Our subsequent targeted validation of a selected group of candidates related to the ubiquitin‐proteasome pathway suggests a mechanism through which E2 via ERα may function as an anti‐catabolic sex hormone in female skeletal muscle.

The ubiquitin‐proteasome system is an important regulator of skeletal muscle protein homeostasis, affecting muscle mass through the crosstalk between anabolic and catabolic pathways [31]. To date, many studies of the ubiquitin‐proteasome pathway in skeletal muscle have involved aged rodents. As reviewed previously, studies on skeletal muscle have revealed mixed results in regard to changes in the levels of ubiquitin ligases (MuRF1 and atrogin), total ubiquitin, as well as in proteasome content and activity with aging [32]. The majority of such studies used only males or did not report sex; the ubiquitin‐proteasome pathway in skeletal muscle of female rodents has received little investigation. Moreover, a limited number of studies have specifically examined the effects of E2 on ubiquitin and proteasome in muscle. In the soleus muscle of young ovariectomized mice, E2 treatment resulted in a decreased level of ubiquitinated proteins [33], consistent with the findings presented here on overall ubiquitination in TA muscle of ovariectomized mice (Figure 5).

We found that pathways and mediators of the ubiquitin‐proteasome system were relatively high in the skeletal muscle of ovariectomized mice, and E2 treatment was repressive (Figures 2, 3, 4). For example, gene or protein levels of proteasome subunits were repressed in the muscle of E2‐treated mice as were ubiquitin‐related genes. In addition to the cascade of enzymes responsible for conjugating ubiquitin to proteins targeting them for degradation, deubiquitinating enzymes called ubiquitin‐specific peptidases (USPs) also moderate the ubiquitin‐proteasome system. By removing ubiquitin from proteins, USPs may rescue them from degradation. Notably, administration of E2 to ovariectomized mice increased gene expression and protein content of deubiquitinating enzyme USP19 in gastrocnemius and soleus muscles [34]. These collective results suggest that E2 may exert beneficial effects on skeletal muscle by suppressing the ubiquitin and proteasome pathways and thus acting as an anti‐catabolic sex hormone in female mice.

Of clinical relevance, a transcriptomic study on skeletal muscle of healthy women at menopause with and without estrogen‐based hormone therapy showed significant regulation of the ubiquitin‐proteasome system indicating catabolic protection [35]. Similarly, gene expression of ubiquitin E3 ligases, MuRF1 and MAFbx, and associated transcription factor FOXO3A was suppressed in muscle from women taking hormone therapy compared with women not on hormone therapy [36]. Furthermore, early postmenopausal women given E2 treatment had decreased protein content of MuRF1 compared with placebo treatment [37]. Consequently, the evidence from the muscle of women indicates that E2‐dependent downregulation of these pathways may be a protective mechanism against atrophy, consistent with our present results in the muscle of female mice.

Overall, a major route of action for E2 in skeletal muscle is through ERα, and here, we explored this notion in view of the ubiquitin‐proteasome system. Our computational analysis revealed that the presence of the putative ERE might play an important role in the repression of the selected genes by E2 (Figure 6). Experiments in skmERαWT and skmERαKO mice with intact ovaries and in ovariectomized skmERαKO mice treated with placebo or E2 suggest that E2 requires ERα to repress the expression of the selected ubiquitin‐proteasome genes in skeletal muscle (Figure 7). Consistent with this idea, ERα knockdown in muscle led to a decrease in the gene expression of deubiquitinating enzyme USP19 [33]. Interestingly, in the aorta and heart, there is a putative repressive role of gene expression for ERβ [38].

A limitation of our study is that the sample size was rather small in some experiments, and the effects of E2 on gene expression were not large. However, small fold changes due to E2 effects have been previously encountered [21, 38]. Further, the consistency of our experimental procedures and data supports the validity of the biological relevance of our findings. It has been previously demonstrated that small quantitative differences in the amount of RNA can have relevant qualitative effects at a functional level in striated muscle [21]. It may also be interesting to measure the effect of E2 on proteasome activity, which was not included in the present experimental design. Other caveats of our study include dose, administration and duration of E2 treatment compared with previous studies [33, 34], which could account for any inconsistent results. Importantly, however, our E2 dosing regimen resulted in serum E2 levels representing physiological levels in mice during proestrus (Figure 1). Further studies manipulating the putative E2 targets identified here will be necessary to investigate downstream effects of E2 on skeletal muscle, particularly on how E2 affects proteasome and ubiquitin‐mediated proteolysis pathways to contribute to the regulation of skeletal muscle mass and ultimately strength. Given that there are sex differences in ubiquitin content in skeletal muscle between young adult male and female mice [33] and that sex‐biased effects of E2 occur in the heart muscle [21, 39], it would be interesting for future studies to directly compare the effects of E2 between the sexes. In addition to sex, other factors, such as specific muscle type, age [7, 40] or genetic background [23], might contribute to differences in E2 responses between muscle from males and females and should therefore also be considered in future studies. Lastly, the inclusion of an ovary‐intact control group would have allowed for insights into E2 potentially mediating ovariectomy‐induced changes back to control/intact levels.

In conclusion, this study provides insight into mechanistic pathways regulated by E2 in skeletal muscle that may be protective against menopause‐related loss of muscle mass and strength. Specifically, regulation of ubiquitin‐proteasome mediators appears to be mediated, at least in part, by the E2/ERα axis in the skeletal muscle of adult females.

## Conflicts of Interest

The authors declare no conflicts of interest.

## Supporting information


**Table S1.** Genes.

**Table S2.** Pathways.

## Data Availability

The data that support the findings of this study are available from the corresponding author upon reasonable request.
