# Supplementary material for: Transcriptomic Profiling Reveals 17β‐Estradiol Treatment Represses Ubiquitin‐Proteasomal Mediators in Skeletal Muscle of Ovariectomized Mice
Source: J Cachexia Sarcopenia Muscle. 2025 Jan 25;16(1):e13698. doi: 10.1002/jcsm.13698 (PMC11761681; doi:10.1002/jcsm.13698)
Supplement: Supplementary file 1 — Table S1. Genes. [file JCSM-16-e13698-s001.docx]

**Supplementary Table 1. Genes**

| **Probe** | **Symbol** | **Name** | **GenBank** | **Gene** | **p-value** | **Fold Change E2 vs Placebo** |
| --- | --- | --- | --- | --- | --- | --- |
| 1418702_a_at | 2810428I15Rik | RIKEN cDNA 2810428I15 gene | NM_025577 | 66462 | <0.001 | 0.78 |
| 1425512_at | Map2k7 | mitogen-activated protein kinase kinase 7 | AW541674 | 26400 | <0.001 | 0.82 |
| 1455314_at | Lpp | LIM domain containing preferred translocation partner in lipoma | BM236111 | 210126 | <0.001 | 0.76 |
| 1456706_at | 4833441D16Rik | RIKEN cDNA 4833441D16 gene | BE628134 | 108962 | <0.001 | 0.77 |
| 1428382_at | Smarcc2 | SWI/SNF related, matrix associated, actin dependent regulator of chromatin, subfamily c, member 2 | AK013190 | 68094 | <0.001 | 0.82 |
| 1446621_at |  |  | AW554535 |  | <0.001 | 0.81 |
| 1432289_a_at | Jsrp1 | junctional sarcoplasmic reticulum protein 1 | AK009016 | 71912 | <0.001 | 0.81 |
| 1423551_at | Cdh13 | cadherin 13 | BB776961 | 12554 | <0.001 | 0.76 |
| 1443161_at |  |  | BM240648 |  | <0.001 | 0.78 |
| 1423592_at | Rock2 | Rho-associated coiled-coil containing protein kinase 2 | BB761686 | 19878 | <0.001 | 0.81 |
| 1422233_at | Elk4 | ELK4, member of ETS oncogene family | NM_007923 | 13714 | <0.001 | 1.18 |
| 1418401_a_at | Dusp16 | dual specificity phosphatase 16 | NM_130447 | 70686 | <0.001 | 0.83 |
| 1436841_at | Fam63b | family with sequence similarity 63, member B | AV229336 | 235461 | <0.001 | 0.77 |
| 1456133_x_at | Itgb5 | integrin beta 5 | BB543646 | 16419 | <0.001 | 0.79 |
| 1448702_at | Ier3ip1 | immediate early response 3 interacting protein 1 | BE287896 | 66191 | <0.001 | 0.81 |
| 1424986_s_at | Fbxw7 | F-box and WD-40 domain protein 7 | AV338062 | 50754 | <0.001 | 0.83 |
| 1433979_at | Rbms2 | RNA binding motif, single stranded interacting protein 2 | BQ176636 | 56516 | <0.001 | 0.84 |
| 1460037_at | Fam98b | family with sequence similarity 98, member B | BF303035 | 68215 | <0.001 | 0.83 |
| 1448822_at | Psmb6 | proteasome (prosome, macropain) subunit, beta type 6 | BC013897 | 19175 | <0.001 | 0.84 |
| 1428620_at | Ensa | endosulfine alpha | BF584204 | 56205 | <0.001 | 0.84 |
| 1435550_at | Mll2 | myeloid/lymphoid or mixed-lineage leukemia 2 | BB702910 | 381022 | <0.001 | 0.78 |
| 1428468_at | 3110043O21Rik | RIKEN cDNA 3110043O21 gene | AK014175 | 73205 | <0.001 | 0.85 |
| 1429461_at | Ints2 | integrator complex subunit 2 | AK013101 | 70422 | <0.001 | 0.86 |
| 1419259_at | Rsu1 | Ras suppressor protein 1 | NM_009105 | 20163 | <0.001 | 0.86 |
| 1456177_x_at | Zfp706 | zinc finger protein 706 | BB288596 | 68036 | <0.001 | 0.81 |
| 1415676_a_at | Psmb5 | proteasome (prosome, macropain) subunit, beta type 5 | NM_011186 | 19173 | <0.001 | 0.84 |
| 1437143_a_at | Tmx1 | thioredoxin-related transmembrane protein 1 | AV310544 | 72736 | <0.001 | 0.82 |
| 1415755_a_at | Ube2v1 | ubiquitin-conjugating enzyme E2 variant 1 | BC019372 | 66589 | <0.001 | 0.83 |
| 1454776_at | Ehmt1 | euchromatic histone methyltransferase 1 | BB409568 | 77683 | <0.001 | 0.85 |
| 1436809_a_at | Spin1 | spindlin 1 | AV100078 | 20729 | <0.001 | 0.86 |
| 1447917_x_at |  |  | BB303908 |  | <0.001 | 0.83 |
| 1449947_s_at | Zfhx3 | zinc finger homeobox 3 | NM_007496 | 11906 | <0.001 | 0.86 |
| 1436956_at |  |  | AV174028 |  | <0.001 | 0.82 |
| 1420628_at | Pura | purine rich element binding protein A | NM_008989 | 19290 | <0.001 | 0.73 |
| 1436342_a_at | Ubxn1 | UBX domain protein 1 | AU041099 | 225896 | <0.001 | 0.76 |
| 1451211_a_at | Eif2d | eukaryotic translation initiation factor 2D | BC025036 | 16865 | <0.001 | 0.85 |
| 1454758_a_at | Tsc22d1 | TSC22 domain family, member 1 | AU016382 | 21807 | <0.001 | 0.82 |
| 1434765_at |  |  | AI844868 |  | <0.001 | 0.86 |
| 1455168_a_at | Gnb2l1 | guanine nucleotide binding protein (G protein), beta polypeptide 2 like 1 | BM210111 | 14694 | 0.001 | 0.84 |
| 1418571_at | Tnfrsf12a | tumor necrosis factor receptor superfamily, member 12a | NM_013749 | 27279 | 0.001 | 1.34 |
| 1416291_at | Psmc4 | proteasome (prosome, macropain) 26S subunit, ATPase, 4 | NM_011874 | 23996 | 0.001 | 0.86 |
| 1425323_a_at | Fam173a | family with sequence similarity 173, member A | BC008155 | 214917 | 0.001 | 0.86 |
| 1428898_at | Mon1a | MON1 homolog A (yeast) | AK013387 | 72825 | 0.001 | 0.86 |
| 1456413_at | Pde4dip | phosphodiesterase 4D interacting protein (myomegalin) | BB235927 | 83679 | 0.001 | 0.71 |
| 1428594_at | Ralgapa1 | Ral GTPase activating protein, alpha subunit 1 | AK009111 | 56784 | 0.001 | 0.85 |
| 1416533_at | Egln2 | EGL nine homolog 2 (C. elegans) | NM_053208 | 112406 | 0.001 | 0.81 |
| 1455958_s_at |  |  | AI881989 |  | 0.001 | 0.86 |
| 1439244_a_at | Tnrc6a | trinucleotide repeat containing 6a | BB822587 | 233833 | 0.001 | 0.84 |
| 1456830_at | Ppp1r2 | protein phosphatase 1, regulatory (inhibitor) subunit 2 | BB542221 | 66849 | 0.001 | 0.84 |
| 1434373_at | Fam168a | family with sequence similarity 168, member A | BB699417 | 319604 | 0.001 | 0.82 |
| 1424111_at | Igf2r | insulin-like growth factor 2 receptor | BG092290 | 16004 | 0.001 | 0.84 |
| 1415816_at | Cct7 | chaperonin containing Tcp1, subunit 7 (eta) | NM_007638 | 12468 | 0.001 | 0.84 |
| 1427442_a_at | App | amyloid beta (A4) precursor protein | BC005490 | 11820 | 0.001 | 0.82 |
| 1456715_at |  |  | BM238052 |  | 0.001 | 0.87 |
| 1419687_at | Macrod1 | MACRO domain containing 1 | NM_134147 | 107227 | 0.001 | 0.82 |
| 1448479_at | Psmd3 | proteasome (prosome, macropain) 26S subunit, non-ATPase, 3 | NM_009439 | 22123 | 0.001 | 0.82 |
| 1450741_at | Stau1 | staufen (RNA binding protein) homolog 1 (Drosophila) | AW537709 | 20853 | 0.001 | 0.86 |
| 1423739_x_at | Aplp2 | amyloid beta (A4) precursor-like protein 2 | U15571 | 11804 | 0.001 | 0.82 |
| 1456176_x_at | Slc25a39 | solute carrier family 25, member 39 | AV209793 | 68066 | 0.001 | 0.87 |
| 1457675_at | 2510002D24Rik | RIKEN cDNA 2510002D24 gene | BG063089 | 72307 | 0.001 | 0.85 |
| 1442408_at | Sulf2 | sulfatase 2 | BE981170 | 72043 | 0.001 | 0.78 |
| 1423200_at | Ncor1 | nuclear receptor co-repressor 1 | U22016 | 20185 | 0.001 | 0.87 |
| 1421829_at | Ak4 | adenylate kinase 4 | NM_009647 | 11639 | 0.001 | 1.14 |
| 1452674_a_at | Eif3k | eukaryotic translation initiation factor 3, subunit K | BI904160 | 73830 | 0.001 | 0.82 |
| 1434412_x_at | Stub1 | STIP1 homology and U-Box containing protein 1 | AW490135 | 56424 | 0.001 | 0.85 |
| 1443849_x_at | Urod | uroporphyrinogen decarboxylase | AV292769 | 22275 | 0.001 | 0.85 |
| 1436420_a_at | Ipo4 | importin 4 | BB390936 | 75751 | 0.001 | 0.84 |
| 1415869_a_at | Trim28 | tripartite motif-containing 28 | NM_011588 | 21849 | 0.001000 | 0.84 |
| 1437468_x_at | Fbxw11 | F-box and WD-40 domain protein 11 | BB315985 | 103583 | 0.001 | 0.85 |
| 1446328_at |  |  | BB470610 |  | 0.001 | 1.14 |
| 1457716_at | Otud7b | OTU domain containing 7B | BM235074 | 229603 | 0.001 | 0.83 |
| 1437985_a_at | 2310061I04Rik | RIKEN cDNA 2310061I04 gene | BB379386 | 69662 | 0.001 | 0.83 |
| 1445944_at |  |  | BG072805 |  | 0.001 | 1.14 |
| 1437080_s_at | Psmd11 | proteasome (prosome, macropain) 26S subunit, non-ATPase, 11 | AV296751 | 69077 | 0.001 | 0.84 |
| 1416483_at | Ttc3 | tetratricopeptide repeat domain 3 | BB833716 | 22129 | 0.001 | 0.78 |
| 1458249_at |  |  | BB771227 |  | 0.001 | 0.86 |
| 1440817_x_at | Zfp771 | zinc finger protein 771 | BB242445 | 244216 | 0.001 | 0.85 |
| 1460424_at | Tmem160 | transmembrane protein 160 | BI411309 | 69094 | 0.001 | 0.85 |
| 1425628_a_at | Gtf2i | general transcription factor II I | AF043220 | 14886 | 0.001 | 0.81 |
| 1448853_at | Synj2bp | synaptojanin 2 binding protein | NM_025292 | 24071 | 0.001 | 0.79 |
| 1455306_at | Fam193a | family with sequence similarity 193, member A | BB473660 | 231128 | 0.001 | 0.84 |
| 1456624_at | Wipi1 | WD repeat domain, phosphoinositide interacting 1 | BI251603 | 52639 | 0.001 | 0.82 |
| 1431109_at | Prr16 | proline rich 16 | AI120070 | 71373 | 0.001 | 1.14 |
| 1455391_at |  |  | BI256061 |  | 0.001 | 0.81 |
| 1429329_at | Cox10 | COX10 homolog, cytochrome c oxidase assembly protein, heme A: farnesyltransferase (yeast) | AK010385 | 70383 | 0.001 | 0.81 |
| 1460004_x_at | Stx6 | syntaxin 6 | BB492711 | 58244 | 0.001 | 0.87 |
| 1433993_at | 4931406P16Rik | RIKEN cDNA 4931406P16 gene | BG067664 | 233103 | 0.001 | 0.77 |
| 1449622_s_at | Atp6ap1 | ATPase, H+ transporting, lysosomal accessory protein 1 | AI316502 | 54411 | 0.001 | 0.82 |
| 1424721_at | Mfap3 | microfibrillar-associated protein 3 | BI661422 | 216760 | 0.001 | 0.84 |
| 1428068_at | Samm50 | sorting and assembly machinery component 50 homolog (S. cerevisiae) | AK003990 | 68653 | 0.001 | 0.85 |
| 1417068_a_at | Ptpn1 | protein tyrosine phosphatase, non-receptor type 1 | BC010191 | 19246 | 0.001 | 0.85 |
| 1415727_at | Apoa1bp | apolipoprotein A-I binding protein | AV017766 | 246703 | 0.001 | 0.87 |
| 1416312_at | Rars | arginyl-tRNA synthetase | NM_025936 | 104458 | 0.001 | 0.84 |
| 1451168_a_at | Arhgdia | Rho GDP dissociation inhibitor (GDI) alpha | BC004732 | 192662 | 0.001 | 0.88 |
| 1455800_x_at | Samm50 | sorting and assembly machinery component 50 homolog (S. cerevisiae) | BB085063 | 68653 | 0.001 | 0.86 |
| 1418572_x_at | Tnfrsf12a | tumor necrosis factor receptor superfamily, member 12a | NM_013749 | 27279 | 0.001 | 1.32 |
| 1429454_at | Gapvd1 | GTPase activating protein and VPS9 domains 1 | BM123170 | 66691 | 0.001 | 0.80 |
| 1427923_at | Zmpste24 | zinc metallopeptidase, STE24 homolog (S. cerevisiae) | BM233793 | 230709 | 0.001 | 0.85 |
| 1456135_s_at | Pxn | paxillin | BB530368 | 19303 | 0.001 | 0.74 |
| 1442710_at |  |  | AV352204 |  | 0.001 | 0.71 |
| 1433871_at | R3hdm1 | R3H domain 1 (binds single-stranded nucleic acids) | BQ174018 | 226412 | 0.001 | 0.88 |
| 1439234_a_at | Tm2d2 | TM2 domain containing 2 | BE200117 | 69742 | 0.001 | 0.82 |
| 1452055_at | Ctdsp1 | CTD (carboxy-terminal domain, RNA polymerase II, polypeptide A) small phosphatase 1 | BB770944 | 227292 | 0.001 | 0.83 |
| 1448305_at | Rab6 | RAB6, member RAS oncogene family | NM_024287 | 19346 | 0.001 | 0.84 |
| 1448521_at | Brd7 | bromodomain containing 7 | NM_012047 | 26992 | 0.001 | 0.88 |
| 1438971_x_at | Ube2h | ubiquitin-conjugating enzyme E2H | BB447627 | 22214 | 0.001 | 0.84 |
| 1454834_at | Nfib | nuclear factor I/B | BI687652 | 18028 | 0.001 | 0.85 |
| 1415740_at | Psmc5 | protease (prosome, macropain) 26S subunit, ATPase 5 | NM_008950 | 19184 | 0.001 | 0.84 |
| 1438366_x_at | Clcn3 | chloride channel 3 | BB144743 | 12725 | 0.001 | 0.80 |
| 1416044_at | Flii | flightless I homolog (Drosophila) | NM_022009 | 14248 | 0.001 | 0.86 |
| 1421463_at | Siglece | sialic acid binding Ig-like lectin E | AF329269 | 83382 | 0.001 | 1.15 |
| 1451083_s_at | Aars | alanyl-tRNA synthetase | BC026611 | 234734 | 0.001 | 0.88 |
| 1452889_at | Lhpp | phospholysine phosphohistidine inorganic pyrophosphate phosphatase | AK009207 | 76429 | 0.001 | 0.87 |
| 1429469_at | A930013F10Rik | RIKEN cDNA A930013F10 gene | BB496937 | 68074 | 0.001 | 0.87 |
| 1429900_at | 5330406M23Rik | RIKEN cDNA 5330406M23 gene | BM241296 | 76671 | 0.001 | 0.73 |
| 1451290_at | Map1lc3a | microtubule-associated protein 1 light chain 3 alpha | BC010596 | 66734 | 0.001 | 0.82 |
| 1416922_a_at | Bnip3l | BCL2/adenovirus E1B interacting protein 3-like | AK018668 | 12177 | 0.001 | 0.80 |
| 1423202_a_at | Ncor1 | nuclear receptor co-repressor 1 | U22016 | 20185 | 0.001 | 0.78 |
| 1451969_s_at | Parp3 | poly (ADP-ribose) polymerase family, member 3 | BC014870 | 235587 | 0.001 | 0.87 |
| 1445395_at |  |  | BB114415 |  | 0.001 | 0.83 |
| 1421743_a_at | Pcbp2 | poly(rC) binding protein 2 | NM_011042 | 18521 | 0.001 | 0.83 |
| 1436949_a_at | Tceb2 | transcription elongation factor B (SIII), polypeptide 2 | AV068352 | 67673 | 0.001 | 0.82 |
| 1456153_at | Ssh2 | slingshot homolog 2 (Drosophila) | BB038915 | 237860 | 0.001 | 0.82 |
| 1437151_at | Usp22 | ubiquitin specific peptidase 22 | AI427806 | 216825 | 0.001 | 0.86 |
| 1434299_x_at | Ift27 | intraflagellar transport 27 homolog (Chlamydomonas) | AI413098 | 67042 | 0.001 | 0.86 |
| 1441581_at | Asb10 | ankyrin repeat and SOCS box-containing 10 | AV233423 | 117590 | 0.001 | 0.81 |
| 1448625_at | Golga2 | golgi autoantigen, golgin subfamily a, 2 | NM_133852 | 99412 | 0.001 | 0.86 |
| 1433997_at | Klhdc10 | kelch domain containing 10 | BM213660 | 76788 | 0.001 | 0.87 |
| 1431925_at | 4933433H22Rik | RIKEN cDNA 4933433H22 gene | AK017043 | 74473 | 0.001 | 1.16 |
| 1434914_at | Rab6b | RAB6B, member RAS oncogene family | BB396668 | 270192 | 0.001 | 0.88 |
| 1422486_a_at | Smad4 | MAD homolog 4 (Drosophila) | AK004804 | 17128 | 0.001 | 0.71 |
| 1435360_at | Zfp651 | zinc finger protein 651 | BB076581 | 270210 | 0.001 | 0.82 |
| 1426242_at | Polr2a | polymerase (RNA) II (DNA directed) polypeptide A | U37500 | 20020 | 0.001 | 0.83 |
| 1453155_at | Tmem50a | transmembrane protein 50A | AK014282 | 71817 | 0.001 | 0.87 |
| 1416113_at | Fkbp8 | FK506 binding protein 8 | NM_010223 | 14232 | 0.001 | 0.88 |
| 1424311_at | Carkd | carbohydrate kinase domain containing | BC019538 | 69225 | 0.001 | 0.86 |
| 1416610_a_at | Clcn3 | chloride channel 3 | NM_007711 | 12725 | 0.001 | 0.78 |
| 1427680_a_at | Nfib | nuclear factor I/B | Y07687 | 18028 | 0.001 | 0.80 |
| 1436165_at | Luc7l2 | LUC7-like 2 (S. cerevisiae) | BI076494 | 192196 | 0.001 | 0.73 |
| 1449283_a_at | Mapk12 | mitogen-activated protein kinase 12 | BC021640 | 29857 | 0.001 | 0.81 |
| 1452680_at | Snrpd2 | small nuclear ribonucleoprotein D2 | BQ043840 | 107686 | 0.001 | 0.86 |
| 1448770_a_at | Atpif1 | ATPase inhibitory factor 1 | NM_007512 | 11983 | 0.001 | 0.84 |
| 1436494_x_at | Trmt1 | TRM1 tRNA methyltransferase 1 homolog (S. cerevisiae) | BF319098 | 212528 | 0.001 | 0.84 |
| 1434117_at | Tceb3 | transcription elongation factor B (SIII), polypeptide 3 | AV264059 | 27224 | 0.001 | 0.85 |
| 1460458_at | Crispld2 | cysteine-rich secretory protein LCCL domain containing 2 | AK019034 | 78892 | 0.001 | 0.82 |
| 1437050_s_at | Angel2 | angel homolog 2 (Drosophila) | AV330655 | 52477 | 0.001 | 0.87 |
| 1450903_at | Rad23b | RAD23b homolog (S. cerevisiae) | BF138887 | 19359 | 0.001 | 0.83 |
| 1417024_at | Hars | histidyl-tRNA synthetase | BC020088 | 15115 | 0.001 | 0.87 |
| 1420709_s_at | Dao | D-amino acid oxidase | BC018377 | 13142 | 0.001 | 1.15 |
| 1436774_at | Sel1l | sel-1 suppressor of lin-12-like (C. elegans) | BI661339 | 20338 | 0.001 | 0.82 |
| 1449934_at | Pura | purine rich element binding protein A | NM_008989 | 19290 | 0.001 | 0.81 |
| 1434956_at | Rnf170 | ring finger protein 170 | AV145226 | 77733 | 0.001 | 0.73 |
| 1418636_at | Etv3 | ets variant gene 3 | BI456953 | 27049 | 0.001 | 1.13 |
| 1435859_x_at | Psmc2 | proteasome (prosome, macropain) 26S subunit, ATPase 2 | AV263662 | 19181 | 0.001 | 0.87 |
| 1415735_at | Ddb1 | damage specific DNA binding protein 1 | NM_015735 | 13194 | 0.001 | 0.85 |
