# Supplementary material for: Transcriptomic Profiling Reveals 17β‐Estradiol Treatment Represses Ubiquitin‐Proteasomal Mediators in Skeletal Muscle of Ovariectomized Mice
Source: J Cachexia Sarcopenia Muscle. 2025 Jan 25;16(1):e13698. doi: 10.1002/jcsm.13698 (PMC11761681; doi:10.1002/jcsm.13698)
Supplement: Supplementary file 2 — Table S2. Pathways. [file JCSM-16-e13698-s002.docx]

**Supplementary Table 2. Pathways**

| **Induced in E2-treated mice** | | |
| --- | --- | --- |
| **ID** | **Name** | ***P* value** |
| X00120 | Primary bile acid biosynthesis | 0.05 |
| X00140 | Steroid hormone biosynthesis | 0.05 |
| X00565 | Ether lipid metabolism | 0.05 |
| X00590 | Arachidonic acid metabolism | 0.05 |
| X00591 | Linoleic acid metabolism | 0.05 |
| X00592 | alpha-Linolenic acid metabolism | 0.05 |
| X00982 | Drug metabolism - cytochrome P450 | 0.05 |
| X04020 | Calcium signaling pathway | 0.05 |
| X04060 | Cytokine-cytokine receptor interaction | 0.05 |
| X04080 | Neuroactive ligand-receptor interaction | 0.05 |
| X04610 | Complement and coagulation cascades | 0.05 |
| X04640 | Hematopoietic cell lineage | 0.05 |
| X04672 | Intestinal immune network for IgA production | 0.05 |
| X04744 | Phototransduction | 0.05 |
| X04930 | Type II diabetes mellitus | 0.05 |
| X04972 | Pancreatic secretion | 0.05 |
| X04974 | Protein digestion and absorption | 0.05 |
| X04975 | Fat digestion and absorption | 0.05 |
| X04976 | Bile secretion | 0.05 |
| X05150 | Staphylococcus aureus infection | 0.05 |
| X05340 | Primary immunodeficiency | 0.05 |

| **Repressed in E2-treated mice** | | |
| --- | --- | --- |
| **ID** | **Name** | ***P* value** |
| X00010 | Glycolysis / Gluconeogenesis | 0.05 |
| X00030 | Pentose phosphate pathway | 0.05 |
| X00051 | Fructose and mannose metabolism | 0.05 |
| X00190 | Oxidative phosphorylation | 0.05 |
| X00230 | Purine metabolism | 0.05 |
| X00310 | Lysine degradation | 0.05 |
| X00510 | N-Glycan biosynthesis | 0.05 |
| X00511 | Other glycan degradation | 0.05 |
| X00514 | Other types of O-glycan biosynthesis | 0.05 |
| X00533 | Glycosaminoglycan biosynthesis - keratan sulfate | 0.05 |
| X00534 | Glycosaminoglycan biosynthesis - heparan sulfate | 0.05 |
| X00600 | Sphingolipid metabolism | 0.05 |
| X00630 | Glyoxylate and dicarboxylate metabolism | 0.05 |
| X00860 | Porphyrin and chlorophyll metabolism | 0.05 |
| X00900 | Terpenoid backbone biosynthesis | 0.05 |
| X00970 | Aminoacyl-tRNA biosynthesis | 0.05 |
| X01100 | Metabolic pathways | 0.05 |
| X02010 | ABC transporters | 0.05 |
| X03008 | Ribosome biogenesis in eukaryotes | 0.05 |
| X03010 | Ribosome | 0.05 |
| X03013 | RNA transport | 0.05 |
| X03018 | RNA degradation | 0.05 |
| X03020 | RNA polymerase | 0.05 |
| X03050 | Proteasome | 0.05 |
| X03060 | Protein export | 0.05 |
| X04010 | MAPK signaling pathway | 0.05 |
| X04012 | ErbB signaling pathway | 0.05 |
| X04110 | Cell cycle | 0.05 |
| X04114 | Oocyte meiosis | 0.05 |
| X04115 | p53 signaling pathway | 0.05 |
| X04120 | Ubiquitin mediated proteolysis | 0.05 |
| X04130 | SNARE interactions in vesicular transport | 0.05 |
| X04141 | Protein processing in endoplasmic reticulum | 0.05 |
| X04142 | Lysosome | 0.05 |
| X04144 | Endocytosis | 0.05 |
| X04150 | mTOR signaling pathway | 0.05 |
| X04260 | Cardiac muscle contraction | 0.05 |
| X04310 | Wnt signaling pathway | 0.05 |
| X04330 | Notch signaling pathway | 0.05 |
| X04380 | Osteoclast differentiation | 0.05 |
| X04520 | Adherens junction | 0.05 |
| X04614 | Renin-angiotensin system | 0.05 |
| X04621 | NOD-like receptor signaling pathway | 0.05 |
| X04622 | RIG-I-like receptor signaling pathway | 0.05 |
| X04660 | T cell receptor signaling pathway | 0.05 |
| X04662 | B cell receptor signaling pathway | 0.05 |
| X04710 | Circadian rhythm - mammal | 0.05 |
| X04720 | Long-term potentiation | 0.05 |
| X04722 | Neurotrophin signaling pathway | 0.05 |
| X04912 | GnRH signaling pathway | 0.05 |
| X04914 | Progesterone-mediated oocyte maturation | 0.05 |
| X04962 | Vasopressin-regulated water reabsorption | 0.05 |
| X05010 | Alzheimer's disease | 0.05 |
| X05012 | Parkinson's disease | 0.05 |
| X05014 | Amyotrophic lateral sclerosis (ALS) | 0.05 |
| X05016 | Huntington's disease | 0.05 |
| X05020 | Prion diseases | 0.05 |
| X05200 | Pathways in cancer | 0.05 |
| X05210 | Colorectal cancer | 0.05 |
| X05211 | Renal cell carcinoma | 0.05 |
| X05212 | Pancreatic cancer | 0.05 |
| X05213 | Endometrial cancer | 0.05 |
| X05214 | Glioma | 0.05 |
| X05215 | Prostate cancer | 0.05 |
| X05216 | Thyroid cancer | 0.05 |
| X05218 | Melanoma | 0.05 |
| X05219 | Bladder cancer | 0.05 |
| X05220 | Chronic myeloid leukemia | 0.05 |
| X05221 | Acute myeloid leukemia | 0.05 |
| X05223 | Non-small cell lung cancer | 0.05 |
